# An insight into the iPSCs-derived two-dimensional culture and three-dimensional organoid models for neurodegenerative disorders

**DOI:** 10.1098/rsfs.2022.0040

**Published:** 2022-08-12

**Authors:** Anushka Bhargava, Ana M. Sandoval Castellanos, Sonali Shah, Ke Ning

**Affiliations:** Sheffield Institute for Translational Neuroscience, Department of Neuroscience, The University of Sheffield, Sheffield S10 2HQ, UK

**Keywords:** two-dimensional cultures, three-dimensional models, organoids, neurodegenerative disorders

## Abstract

The use of induced pluripotent stem cells (iPSCs) is a promising approach when used as models to study neurodegenerative disorders (NDDs) *in vitro*. iPSCs have been used in *in vitro* two-dimensional cultures; however, these two-dimensional cultures do not mimic the physiological three-dimensional cellular environment. The use of iPSCs-derived three-dimensional organoids has risen as a powerful alternative to using animal models to study NDDs. These iPSCs-derived three-dimensional organoids can resemble the complexity of the tissue of interest, making it an approachable, cost-effective technique, to study NDDs in an ethical manner. Furthermore, the use of iPSCs-derived organoids will be an important tool to develop new therapeutics and pharmaceutics to treat NDDs. Herein, we will highlight how iPSCs-derived two-dimensional cultures and three-dimensional organoids have been used to study NDDs, as well as the advantages and disadvantages of both techniques.

## 1. Introduction

Neurodegenerative disorders (NDDs) are chronic and progressive diseases, characterized by the loss of function in sensory, motor or cognitive systems, impairing the central nervous system (CNS) and the peripheral nervous system (PNS), via the loss of neurons and neuronal subtypes [1–3]. Amyotrophic lateral sclerosis (ALS), Alzheimer's disease (AD), Parkinson's disease (PD), spinal muscular atrophy (SMA), multiple sclerosis (MS) and Huntington's disease (HD) are some of the NDDs that affect the global population [2]. The mechanisms behind NDDs are not clearly understood, even though it is known that ageing, protein misfolding, genetics and apoptosis are involved in the process behind NDDs [1,2]. A significant and growing portion of the global health burden is caused by neurological illnesses. In 2017, there were 1.1 million fatalities overall in the EU and 1.97 million overall in the WHO European area, with 21 million disability-adjusted life-years related to neurological illnesses in the EU and 41 million in the WHO European region. In the EU, neurological illnesses are the third most frequent cause of disability and early mortality, and as the population of Europe ages, it is probable that both their frequency and burden will rise [4]. Therefore, health officials need to focus more on treating and preventing neurological illnesses, and the majority of economic and scientific resources are set on tackling such conditions. The foremost challenge in understanding the molecular mechanisms of NDDs is presented within the inability of *in vitro* models to successfully mimic the phenotypic physiology of NDDs. Therefore, it is difficult to develop appropriate drugs to alleviate patient symptoms, and maybe, slow the progression of the NDDs [2,5].

Animal models have been used to study NDDs, as they provide an understanding of the pathogenesis and pathobiology of NDDs. Nevertheless, these models are limited because these models usually overexpress mutant proteins, which affect the pathology of the animal and hence obscure the understanding of the onset and progression of NDDs [2,6,7]. Furthermore, the use of animal models raises ethical considerations regarding their use in research. Russel & Burch established, in 1959, the principle of the 3Rs, which focuses on solving these ethical considerations by **R**educing the number of animals used per experiment, **R**efining methods to increase the welfare of animals and **R**eplacement methods that aim to avoid and replace the use of animal in experiments [8,9]. Therefore, to follow the 3Rs principle, novel models need to be developed and used to further characterize the pathophysiology of NDDs, leading to a path were these models could also be used for the development of therapeutics. In addition, the limitations of animal models are due to the differences and variabilities between different species [10–14]. Besides, the use of human tissue to study NDDs is restricted by the inability to obtain either brain or spinal cord tissue from NDD patients throughout the disease [2,15,16]. Therefore, it is of high importance to develop models that mimic the progression of NDDs to obtain a better understanding of the onset and development of NDDs. The use of induced pluripotent stem cells (iPSCs) is a promising alternative to model and study NDDs *in vitro* [17,18]. iPSCs can be obtained from the reprogramming of somatic cells, such as fibroblasts and peripheral blood, from healthy and patient donors [15,19]. Furthermore, as iPSCs have self-renewal properties and can differentiate into any cell type, iPSCs can differentiate into motor neurons, oligodendrocytes, astrocytes and microglia. Therefore, iPSCs can be differentiated to neuronal cells and can be used to study and understand the onset and progression of various NDDs [15,18–20].

iPSCs have been used in *in vitro* two-dimensional cultures; however, these two-dimensional cultures do not mimic the physiological three-dimensional cell environment, as two-dimensional cultures lack nutritional, waste and oxygen gradients, and interactions among cells or cells between the extracellular matrix (ECM) [10,13]. Hence, three-dimensional organoids (a three-dimensional multicellular construct that resembles a tissue or an organ), using iPSCs have been developed to mimic the *in vivo* environment [2,13], mimicking cell-to-cell and cell-to-ECM interactions, showing cellular growth, proliferation, differentiation, migration, protein production and gene expression [2,18,21–23]. These three-dimensional organoids developed to study NDDs are composed of a heterogeneous population of neural cells, obtained from differentiating iPSCs into motor neurons, astrocytes, and oligodendrocytes [17,18,24]. These three-dimensional organoids are a promising alternative to understanding the onset and progress of NDDs. Furthermore, these models can be used to develop novel therapeutics for NDDs [2]. In this review, we discuss how iPSCs-derived two-dimensional cultures and three-dimensional organoids models have been used to study NDDs and the advantages and challenges of both models.

## 2. Modelling NDDs in iPSCs-derived two-dimensional cultures

The complexity of cellular models of NDDs ranges from simple monolayers (two dimensional) derived from immortalized cell lines to intricate multicellular organoids (three dimensional) which mimic tissue. These are able to replicate many disease hallmarks and *in vivo* physiological conditions. In this section, NDDs like AD, PD, ALS and HD are briefly defined and discussed to highlight the use of iPSCs-derived two-dimensional cultures to model these diseases.

### 2.1. Alzheimer's disease

AD is the most prevalent NDD, characterized by the dense accumulation of beta-amyloid (Aβ) plaques and neurofibrillary tangles, which are defined by the intraneuronal presence of microtubule tau [25]. AD patients suffer from cognitive deficits and memory loss as a result of neuron damage, prominently occurring within the medial temporal lobe and the regions of the neocortex [26]. Environmental factors and inheriting the E4 allele of the apolipoprotein E (APOE *ε*4) present an increased risk of developing sporadic AD. Unlike the APOE *ε*2 and APOE *ε*3 alleles, APOE *ε*4 proteins are far less effective in the breakdown of Aβ plaques [27]. Familial AD occurs with early onset (less than 60 years of age) and is caused by mutations occurring within the amyloid precursor protein (APP), presenilin 1 (PSEN 1), and presenilin 2 (PSEN 2) genes. Both forms of AD present clinical symptoms including gradual memory loss, agnosia and apraxia [25,26].

The mechanisms that cause AD are poorly understood. However, ground-breaking research models have provided insight into AD pathology, over the years. For example, inhibiting acetylcholinesterase (AChE) to increase acetylcholine production has induced the regeneration of neuronal and cognitive activity in AD patients [28]. Additionally, overexpression of *N*-methyl-d-aspartate (NMDA) is known to stimulate an excessive influx of Ca^2+^, leading to excessive production of glutamate. This overexpression of glutamate causes excitotoxicity, neuronal death and deficits in cognitive function [29].

The Food and Drug Administration (FDA) has approved drug-based therapies to treat acetylcholine production and NMDA overexpression [30]. For example, donepezil, rivastigmine, galantamine (AChEIs) and memantine, an NMDA antagonist [26,31]. Tacrine, the first FDA-approved AChEI for AD, was removed from the market as it caused hepatotoxicity in patients [32]. According to Alzheimer's Disease International, the current annual cost of $1 trillion to treat dementia, is estimated to double by the year 2030 [33]. Therefore, it is essential to implement effective use of resources and facilities to tackle the condition.

The two-dimensional tissue culture model is a well-known technique, consisting of a cell monolayer, that has been used since 1907. The iPSCs-derived two-dimensional model involves seeding directly on a glass or polystyrene surface that is coated with agents that support cell adhesion, proliferation, and differentiation. iPSCs-derived two-dimensional models are widely used to study and decipher various neurological conditions including AD [34].

Wang *et al*. [35] reported increased production of Aβ and p-tau levels within hiPSC-originated APOE *ε*4 neurons. The two-dimensional-based study also reported GABAergic neuron degeneration from the APOE *ε*4 variant. However, post-conversion of APOE *ε*4 to isogenic apolipoprotein *ε*3 (APOE *ε*3) using ApoE structural corrector PH002, eliminated the pathogenic behaviour. This not only confirmed the pathogenic activity of APOE *ε*4 linked to AD but also highlighted a new approach to targeting APOE ε4-related AD [35]. This study is a prime example of how combining two-dimensional modelling directly with iPSCs can eliminate obstacles such as species variance as these cells are directly obtained from humans in an ethical manner. The technique also minimizes the use of animals, for example, mice models do not exhibit APOE *ε*4 due to species differences and are also far more cost-effective [35].

Table 1 shows other examples of *in vitro* studies to model AD, where disease-related mechanism and phenotypes were analysed.

**Table 1.** iPSC-derived *in vitro* studies to model AD and analyse disease-related mechanisms and phenotypes.

AD patient-derived iPSCs: genetic mutation	differentiation into cell type	differentiation marker	result	references
PSEN1 mutation A246E	basal forebrain cholinergic neurons	Tuj-1, amyloid-beta	an increased amyloid-beta 42 : 40 ratio	[36]
(APP)-E693D mutation	neurons and astrocytes with an accumulation of amyloid-beta oligomers	Tuj-1, GFAP, amyloid-beta	the accumulation of amyloid-beta oligomers in neurons and astrocytes	[37]
V717I mutation	neurons with reduction of total tau	Tuj-1, amyloid beta, tau	increases in APP expression and reduction of total tau	[38]
APP variant (A673T)	neurons with reduction of amyloid-beta	Tuj-1, amyloid beta,	neurons reduced levels of amyloid-beta	[39]
PS1 (A246E) and PS2 (N141I) mutations	neuron with elevated amyloid-beta 42 : 40	Tuj-1, amyloid beta	significant increase in expression of amyloid beta	[40]
PSEN1 mutation	neuron with increased amyloid-beta 42/40	Tuj-1, amyloid beta	significant increase in expression of amyloid beta	[41]

### 2.2. Parkinson's disease

PD is another lethal NDD in which motor function is impeded. It is the second most prevalent neurological disorder after AD and affects over 7 million people worldwide [42]. Dopaminergic neurons present within the substantia nigra pars compacta are targeted and damaged by multifactorial mechanisms including *α*-synuclein aggregation and misfolding, mitochondrial apoptosis and excitotoxicity [43]. The disorder is late-onset and exists in two forms, the idiopathic form, and the rare familial version. Both forms convey the symptomatic features of bradykinesia, tremor and muscular rigidity [44]. Although the familial form only accounts for 10% of all PD cases, gene-linked PD harbours multiple causative mutations in the *α*-synuclein gene, Leucine-rich repeat kinase 2 (LRRK2) and PTEN-induced kinase 1 (PINK1) genes [44]. The LRRK2 is a vital protein involved in autophagy and maintaining cellular functionality. Mutant LRRK2 is known to alter the autophagy process and has been linked to increased *α*-synuclein aggregation [45]. Therefore, analysing the molecular pathways encoded by these mutations using the two-dimensional model can provide insight for novel treatment. Currently, treatments and therapy options are available that aid in treating PD [46,47].

Despite the plethora of challenges, iPSCs appear to be a promising platform due to their ability to differentiate into midbrain dopaminergic (DA) neurons. Furthermore, using iPSC-related co-cultures, to examine and expand our knowledge relating to PD-associated cell–cell interaction within the brain, appears to be a promising platform. The Kikuchi *et al*. [48] study reported improved motor function in primates, who had undergone surgery to graft DA neurons derived from hiPSCs. Using the dual SMAD inhibition technique, dopaminergic neurons were generated from eight iPSCs cell lines including four healthy individuals and three PD patients, and a single HD patient. Day 28 cultured DA neurons were then grafted into the putamen of cynomolgus monkeys. The study confirmed good recovery rates among both PD and healthy derived cell lines. PD symptoms including dyskinesias were absent from monkeys transplanted with PD-derived neurons. Furthermore, the efficacy of l-DOPA was also tested. Upon administration of l-DOPA, the MPTP-treated monkeys exhibited 15–33% improvement in their PD scores [48]. Although this is a fascinating concept, further research is required as it is in the early stages of development. However, it opens a gateway to using iPSCs-based tissue grafts, to replace the valuable cellular functionality of damaged DA neurons that have previously undergone neurodegeneration. The prospect also provides an accurate insight into targeted drug efficacy and testing as the host is a multicellular living organism [48].

Furthermore, di Domenico *et al*. [42] designed a neuron–astrocyte co-culture to analyse the signal between the midbrain dopaminergic (mDA) neurons and astrocytes derived from mutant LRRK2-iPSCs. By contrast to their healthy controls, the mutant G2019SLRRK2 mDA-astrocyte model recapitulated the typical PD phenotype [42]. This includes the accumulation of α-synuclein, premature cell death, and morphological alterations including loss of membrane within the mDA neurons. Astrocytes possess a neuroprotective role in cells including debris clearance, inflammation stimulation and alleviation of glutamate-related excitotoxicity [49]. However, di Domenico's study showed that upon co-culturing PD-derived astrocytes with healthy control neurons, the astrocytes contributed toward the neurodegeneration of the healthy dopaminergic neurons. α-Synuclein formation was witnessed within the healthy control neurons [42]. The study is a prime example of how two-dimensional culture has aided in discovering crucial information. It has enabled the research community to better understand the aetiology of PD, to treat it accordingly. Table 2 highlights iPSC-derived *in vitro* studies that have modelled and analysed disease-related mechanism and phenotypes of PD.

**Table 2.** iPSC-derived *in vitro* studies to model PD and analyse disease-related mechanisms and phenotypes.

PD patient-derived iPSCs: genetic mutation	differentiation into cell type	differentiation marker	result	references
Parkin	midbrain dopaminergic neurons	Parkin	elevated transcription of monoamine oxidases, inducing oxidative stress	[50]
LRRK2	dopaminergic neurons	dopamine LRRK2	the increased generation of alpha-synuclein protein and increased expression of oxidative stress-response genes	[51–54]
SNCA triplication mutation	midbrain dopaminergic neurons	alpha-synuclein	aggressive form of PD with dementia	[55–57]
glucocerebrosidase and SNCA mutation	functional loss of glucocerebrosidase in iPSC-derived neurons	glucocerebrosidase	the pathogenesis of sporadic synucleinopathies including idiopathic PD	[50]
SNCA triplication	accumulation of alpha-synuclein in iPSC-derived neurons	peroxide	a major phenotype of PD including oxidative stress	[55–57]

### 2.3. Amyotrophic lateral sclerosis

ALS is a progressive NDD through which motor function is impeded via deterioration of the upper and lower motor neurons located in the brainstem and spinal cord, respectively [58]. The adult-onset NDD usually targets those aged over the age of 55. However, the younger population can also be affected [59]. ALS is characterized in two forms, sporadic ALS (SALS) and familial ALS (FALS). The sporadic version accounts for 90–95% of all ALS cases. By contrast, FALS accounts for 5–10% of all ALS cases [60]. Both FALS and SALS victims tend to suffer from a multitude of clinical symptoms including focal limb weakness, deterioration and fatigue. Muscular weakness tends to start with the distal limb muscles rather than the proximal regions [58]. However, throughout degeneration, muscular atrophy remains prominent. Twenty-five to 30% of ALS cases experience bulbar-onset symptoms including dysphasia dysarthria, and some patients experience masseter weakness [58]. Nevertheless, in later stages of ALS, victims experience paralysis and tend to die, with respiratory failure being the ultimate cause of mortality [61]. Consequently, life expectancy for ALS patients is 3–5 years [62]. The common mutant genes associated with FALS include TAR DNA-binding protein 43 (TDP-43), superoxide dismutase 1 (SOD 1), and chromosome 9 open reading frame 72 (C9orf72) [63].

Four per cent of all FALS cases are linked to TDP-43 mutations [64]. TDP-43 is a ubiquitous protein that is encoded by the TARDBP gene. Normal functioning TDP-43 proteins are responsible for a variety of RNA regulatory mechanisms including splicing and transcriptional maintenance and maintaining mRNA stability [65]. Similar to amyloid-β and α-synuclein (pathological hallmarks of AD and PD), TDP-43 also contains prion-like domains, responsible for protein folding and solubility [64]. However, post-translational alterations and mutations in TDP-43 cause these regions to produce TDP-43 proteinopathies [66]. Although the mechanism of TDP-43 pathogenesis is yet to be deciphered, studies have reported that TDP-43 oligomers can successfully promote endogenous TDP-43 aggregations in neighbouring cells via the protein seeding mechanism [64]. Although TDP-43 propagation from cell to cell remains unclear, spread through exomes and tunnelling nanotubes (TNT) appear to be promising concepts of TDP-43 propagation [67]. The Ding *et al*. [67] study combined U251 cells with media containing 30% CFS (extracted from ALS victims). On day 21, immunofluorescence confirmed co-localized TDP-43 seeding, and aggregations were observed in the cells and within TNT-like structures [68].

Of all three mutant genes, through the identification of the repeat expansion GGGGCCC in intron 1, the C9orf72 gene was identified as the most common causative gene of ALS in 2011. The C9orf72 mutation is known to affect 40% of all FALS patients [69]. The GGGGCCC expansion usually ranges from 30 or fewer repeats in healthy individuals. However, in pathological ALS and frontal dementia patients, the repeats range from 100–1000 [68]. Bidirectional transcribing of the repeat GGGGCCC expansion generates RNA repeats, in turn leading to the production of RNA foci [70]. Additionally, non-ATG translations of the repeat RNA enable the successful generation of dipeptide repeat proteins (DPRs) [71]. The three main pathogenic mechanisms associated with C9orf72-induced ALS include DPR production, haploinsufficiency, and C9orf72 protein function loss and gain of toxic function due to C9orf72 RNA repeats. Additionally, RNA binding proteins (RBPs) play a vital role in RNA splicing, translation, processing, and maintenance. Generation of neurotoxic RNA foci has been known to sequester RBPS. Therefore, the downstream signalling pathways of functional RNA processes are affected and contribute toward motor neuron degeneration [72]. As several ALS-related mutations exist, there may be a multitude of pathogenic drivers that impact various cellular pathways, rather than a sole singular causal entity [73]. Accordingly, further research is required to understand ALS pathogenesis and tackle the condition.

Due to species variances, *in vivo* animal models have failed in capturing ALS-related phenotypic recapitulation and drug efficacy has failed during the clinical trial stages [74]. Conversely, iPSCs have thrived in this sector as they are directly derived from the human lineage. Multiple iPSC *in vitro* models have conveyed ALS-associated pathways and phenotypes including TDP-43 pathology, C9orf72 toxicity in neurofilaments. *In vitro* iPSC-generated models in which gene-specific phenotypes linked to FALS have been generated, are shown in table 3.

**Table 3.** iPSC-derived *in vitro* studies to model ALS and analyse disease-related mechanisms and phenotypes.

ALS patient-derived iPSCs: genetic mutation	differentiation into cell type	differentiation marker	result	references
C9orf72	motor neurons	Tuj-I	C9orf72 DPR aggregation	[75]
			C9orf72 RNA Foci	
			glutamate excitotoxicity	
			hyperexcitability	
TDP-43	motor neurons	Isl- I	TDP-43 aggregation	[76]
		Tuj-I	cytoplasmic granules	
			short neurites	
TDP-43	motor neurons	HB9	immature neurite growth	[77]
SOD-1		ChAT	action potential irregularities	
C9ORF72		SMI-32	SOD1 aggregation	
			autophagy dysfunction	

Furthermore, the Kim *et al*. [78] study used the CRISPR/Cas9 genome editing system to introduce SOD1-G93A missense mutation within iPSCs. They successfully reported ALS phenotypes within motor neurons derived from the edited iPSCs. The phenotypes included SOD1 protein accumulation within cells, axonal swelling, and shortened axonal bodies with abnormal structural morphology [79].

### 2.4. Huntington's disease

HD is a hereditary neurodegenerative disorder with expansion of CAG repeats in huntingtin (Htt) [80,81]. CAG causes the degeneration of the GABAergic projection neurons in the striatum regions and the development of involuntary movement and psychiatric disturbance [80–83]. There is no effective of treatment for HD. Though the disease mutant, mtHtt, was discovered two decades ago, the mechanism of HD neurodegeneration remains unclear. Due to the strong correlation between the CAG length and HD, establishing a disease model using iPSCs would be ideal. Zhang *et al.* [79] were among the early authors to generate an iPSC-derived HD model. They developed iPSCs from HD patients displaying CAG repeats and then generated striatal neurons susceptible to cellular damage with typical characteristics of HD, such as mHTT aggregation and decreased concentrations of glutamate transporters and BDNF [80–84]. Their results showed an increased caspase activity upon growth factor deprivation, demonstrating the suitability of the HD iPSC-derived neurons for drug screening. After that, more and more two-dimensional and three-dimensional cell models have been generated in the past two decades (table 4).

**Table 4.** iPSC-derived *in vitro* studies to model HD and analyse disease-related mechanisms and phenotypes.

HD patient-derived iPSCs: genetic mutation	differentiation into cell type	differentiation marker	result	references
180 HTT CAG repeats	cortical neurons	Tuj-I	reduction in pS202 levels in differentiated cortical neurons	[80]
Q47	Q47 striatal neurons	Tuj-I	reduction of the formation of VCP-LC3-mHTT ternary complex	[81]
MSNs	striatal medium spiny neurons (MSNs), microglia	Map2, TREM2 and IBA1	striatal neurons with DARPP32^+^ neurons	[82,83]
	GABA+ MSNs	Map2, GABA	DARPP32 positivity; increased caspase activity	[84,85]
	TUJ1+, MAP2+ and Olig2+ neurons	TUJ1, MAP2,	ARPP-32 positivity; higher rate of DNA damage	[86]
	TUJ1+, MAP2+, neurons	TUJ1, MAP2,	elevated levels of caspase activity upon growth factor withdrawal	[87]
	GABAergic neurons	GABA	hiPSCs generated mostly GABAergic neurons	[88]

### 2.5. Challenges of using two-dimensional models

Two-dimensional models are useful tools in NDDs evaluation as they are easy to manage, highly cost-effective, require fewer ethical considerations, and do not require the subject to be compromised, unlike *in vivo* animal models [89].

Two-dimensional models are unable to mimic the *in vivo* microenvironment of the human brain as in two-dimensional models, cellular processes occur on a flat monolayer surface, as opposed to the three-dimensional direction of the brain [90,91]. *In vitro* models lack the organization and complexity and architecture of the brain. The brain consists of various cell subtypes and multiple molecular mechanisms occur instantaneously. Although two-dimensional co-cultures are available, establishing this level of complexity remains a great challenge [91].

Additionally, cell–cell interactions are limited within the monolayer culture as they only occur on a side-side basis [92]. Cell–matrix interactions are also absent within the *in vitro* two-dimensional culture model. The interactions are essential for processes including cellular proliferation, protein and gene expression, cellular differentiation, drug metabolism and other cellular functions [34]. These processes are directly affected and therefore the *in vitro* two-dimensional culture model does not accurately represent the *in vivo* cellular physiology, characteristics, and molecular mechanisms of the brain and central nervous system [91].

Furthermore, the flattened cell morphology and artificially induced polarity have been reported to alter normal cellular functions including apoptosis and other biochemical pathways [93]. Therefore, analysing the true nature of NDDs is restricted within these models. Due to differences in morphology and organization, two-dimensional cells possess elevated sensitivity when targeted with drug therapies [94]. Even though many two-dimensional models reach the pre-clinical trial stages, translation and applicability to the *in vivo* setting often fail. Despite the plethora of benefits, two-dimensional culture models have exhibited difficulty in unveiling the pathology and pathogenesis of NDDs.

## 3. Modelling NDDs using iPSCs-derived three-dimensional organoids

To overcome these obstacles, using iPSCs-derived three-dimensional organoids may be the frontier technique to recapitulate complex multicellular physiology. Three-dimensional organoids are small, self-organized three-dimensional multicellular tissue cultures that are grown *in vitro*. Each specific organoid can mimic its corresponding organ, such that the cultures can replicate the complexity of an organ *in vivo* and can be used to study selected characteristics of that organ in an *in vitro* culture. Three-dimensional organoids are commonly derived from stem cells and the process that forms organoids *in vitro* can be similar to natural development [95]. Starting from stem cell-derived complexes *in vivo*-like structures, researchers have now grown organoids that model several organs, including the brain, kidney, liver and retina [24,96–99].

The human brain is a complex organ, and its complexity has made it challenging to study various brain disorders in animal models. This highlights the need to develop an *in vitro* model of the human brain. In 2013, the first ever three-dimensional organoid of the brain was successfully generated which possessed cortical-like self-organizing regions (resembling the structure present in the early neuronal development stage), contained functional neurons, and possessed an unformed choroid plexus and retina [24]. They also generated dorsalized neuroepithelium. The three-dimensional organoid closely mimicked both the functionality and structure of a real brain. Furthermore, the three-dimensional organoids also exhibited basal radial glial cells which are lacking in mice models. In addition to the cerebral three-dimensional organoids recapitulating features of human cortical development, here they also modelled microcephaly, a disorder that has been challenging to recapitulate in mice. The optimized methodology presented within the study opened a research gateway that has led to the development of the hippocampus, astrocytes and introduces gyri-like folding. This study reiterated the importance of organoids as they can show development and disease in a complex microenvironment, even in the most complex human organ.

The emerging three-dimensional organoid technology, which produces a model system that better reflects the human brain microenvironment, has been widely used to investigate the development and disorders of the human brain. The development of brain three-dimensional organoids is similar to that of a human fetal brain. This enables the three-dimensional organoids to be used to model neurodevelopment disorders. As an example to emphasize the importance of developing brain three-dimensional organoids to study diseases, human iPSCs-derived three-dimensional organoids have been used to demonstrate the cellular tropism and pathogenesis of the Zika virus [100,101]. Another example is that the occurrence of changes in the fetal brain, when exposed to severe environments, can be mimicked in brain three-dimensional organoids. Additionally, a study highlighted the use of brain three-dimensional organoids to better understand hypoxic encephalopathy of prematurity that results from a severe hypoxic–ischaemic episode [102]. Thus, these three-dimensional organoids models highlights the importance of continuing to use this three-dimensional organoid technology to understand different disorders, such as NDDs.

With NDDs proving challenging to recapitulate in animal models and in traditional two-dimensional cell cultures, iPSCs-derived three-dimensional organoids can be a better alternative to bridge the gaps between our understanding of NDDs in animal models and human patients. iPSC-derived three-dimensional organoids are being developed to study neurodegenerative disorders. Pamies *et al*. developed an *in vitro* iPSC-derived three-dimensional organoid, composed of mature dopaminergic, glutamatergic and GABAergic neurons, oligodendrocytes and astrocytes. They were able to detect axon myelination and electrical activity [13]. A few examples of iPS-derived three-dimensional organoids in each of the common NDDs have been listed in table 5.

**Table 5.** iPSC-derived three-dimensional organoids to model NDDs, which display disease-related phenotypes and mechanisms.

neurodegenerative disorders	model and mutation	structures replicated	differentiation marker	references
ALS	motor neuron organoid	an organoid from iPSC-derived neurons	motor neuron: ChAT, HB9, SMI-32	[103]
	ALS and sensorimotor organoid	sensorimotor organoids containing functional human NMJs	nerve: neurofilament or SV2/Thy1 muscle: AChRs (α-bungarotoxin)	[104]
AD	neurons and astrocyte with pathological accumulation of amyloid-beta	Tuj-I, amyloid-beta, GFAP	showing pathological accumulation of amyloid-beta peptides	[25,105–107]
	cortical organoids	APP duplication; PSEN1 M146I; PSEN1 A264E	amyloid aggregation; hyperphosphorylated tau protein; endosome abnormalities	[108]
PD	LRRK2-G2019S neuron organoid	LRRK2 dopaminergic neurons	three-dimensional midbrain PD organoids to mimic the age-induced modelling of PD	[78,109]
	midbrain organoids	dopaminergic neurons, oligodendrocytes and astrocytes	midbrain organoids replicate neurotoxin-based PD multiple brain regions	[110]
	Parkinson's disease multisystem organoid	dopaminergic neurons	organoids with distinct expression profiles of genes associated with synaptic transmission	[111]
	neuroectodermal spheres	LRRK2 (G2019S) neurons	organoids with DARPP32^+^ neuros	[112]
HD	striatal organoids	Map2, ARPP32	organoids with neurons with CAG repeats over 100	[82,113]
	neurons with CAG repeats (Q109 and Q180)	Map2, GABA Tui1	organoids with neurons with CAG repeats	[114]
	neurons with CAG repeats	Map2, Tui1	organoids with neurons with CAG repeats	[115]

### 3.1. Benefits and limitations of iPSC-derived three-dimensional organoids

Named ‘Method of the year 2017’ by *Nature Methods*, three-dimensional organoid models present a powerful tool to study organ development, pathologies and facilitate therapeutic applications [116]. Three-dimensional organoids are able to more closely mimic *in vivo* tissues than the existing two-dimensional models for research [10,13]. *In vivo*, the cells are part of a microenvironment where they are exposed to various signalling interactions, which are important for regulating the effective function of the tissue as well as maintaining phenotypes. Two-dimensional organoids successfully allow for cell–cell and/or cell–ECM interactions, while the interactions of cells in a two-dimensional model are limited. Cells in the two-dimensional model are fairly uniform and can represent just one cell type due to exposure to a consistent concentration of factors in media. While this allows for studying cellular processes and disease mechanisms in a specific affected cell type, it does not correctly represent cells in human tissue, which are exposed to a concentration gradient of signalling factors, nutrients and crosstalk with other cell types. This is achieved to an extent by three-dimensional organoids, where the outer layer of cells is exposed to a higher concentration of factors compared to the cells at the centre of the sphere-like three-dimensional organoid. This allows for different populations of cell types to proliferate and differentiate in a single three-dimensional organoid model, which closely resembles conditions *in vivo*. Furthermore, not only different cell types could be achieved, but structures. For example, gyrification, known as surface folding, of the human cerebrum is the foundation for the advanced cognitive abilities of humans. It enables a substantial sum of neurons in a small volume. Recently, a study in 2017 modelled the growth and structural formation of the human cortex *in vitro* using cerebral three-dimensional organoids [117]. Gyrification and expansion were achieved through enhancing the PTEN-AKT pathway. With further advancements to this technique, larger brain three-dimensional organoids could be achieved with a gyrification pattern similar to human brains. As mouse models used to study various brain disorders and development do not have a gyrification process, the use of brain three-dimensional organoids could enhance our understanding and bridge this gap in our knowledge. For these reasons, three-dimensional organoids may be a physiologically relevant reductionist model of *in vivo* biology to recapitulate therapeutic applications that have been optimized and show effective outcomes in two-dimensional cultures but have been challenging to replicate in *in vivo* models and human clinical trials. Nevertheless, apart from this study, three-dimensional organoids are currently unable to completely recapitulate the higher complexity of the human brain and the reliability of these models should be considered when identifying developmental processes and cell-specific human brain disorders.

Three-dimensional organoid technology has led to advances in the research of brain development, modelling NDDs and provides a promising medium for testing and developing therapeutic strategies; however, this technology is still in its infancy. An extended period of growth of brain three-dimensional organoids can generate a broad diversity of cells [118]. As mentioned before, iPSCs from brain three-dimensional organoids can be differentiated into diverse neuronal cell types, for example, motor neurons and astrocytes. Nevertheless, cellular diversity recapitulated in brain three-dimensional organoids remains limited. Brain three-dimensional organoids from human iPSCs lack brain-resident macrophages, called microglia, which act as the active immune barrier in the brain, as well as vascular cells and therefore the blood–brain barrier [119]. iPSC-derived organoids can form many neuronal subtypes; however, as the neuroprogenitor ancestor of iPSCs is different from that of microglia (derived from a mesodermal lineage), iPSCs cannot differentiate into microglia [120]. As non-neuronal features of the human brain are largely missing from the three-dimensional organoid culture, they are unable to correctly model brain functions and disorders that involve the interactions between non-neuronal cells or between neuronal and non-neuronal cells. While this is a current limitation, a study has reported cerebral organoids that can innately develop microglia and display their morphology when there is an absence of dual-SMAD inhibition [120]. Another study reported a co-culture system of two-dimensional microglia-like cells with cerebral organoids that could assist to investigate interactions between microglia and neurons [121].

Another limitation of brain iPSCs-derived and all of the other three-dimensional organoids, is that they rely on simple diffusion from the culture for the supply of nutrients and oxygen due to a lack of vascular system with blood vessels. When culturing three-dimensional organoids over a long period of time, a significant number of cells in the centre of the organoids may undergo apoptosis due to a lack of access to oxygen and nutrients. To successfully recapitulate a human brain, an improved circulation system needs to be established for prolonged *in vitro* three-dimensional organoid cultures. Although vascularisation has been achieved using xenotransplantation in mice, the three-dimensional organoid as a single entity is devoid of vasculature and cannot exist without the use of symbiosis with the host [122]. Recently, human blood vessel three-dimensional organoids have been developed from iPSCs that contained endothelial cells and pericytes that are capable of self-assembling into capillary networks. When transplanted into mice, these organoids formed a stable, perfused vascular tree, faithfully resembling the structure and function of human blood vessels [123]. These organoids could be coupled with brain three-dimensional organoids to develop a closed vascular system that can support long-term culture as well as aid in studying neurovascular interactions.

In addition, in comparison to two-dimensional models, three-dimensional NDDs models present higher variability and therefore reproducibility is of concern, which should be carefully considered. Batch variations between each brain organoid such as differences in the composition of cell types can cause variations with experimental results and therefore their interpretation. Despite using the same source of cells, iPSCs most likely, and the same protocols to form three-dimensional organoids in a single batch, due to unpredictable differences in structural compositions and integrity of each of those three-dimensional organoids, they have been known to produce variable and inconsistent results [124].

Though current methods for three-dimensional organoid generation are prone to variable results, a recent study showed the generation of highly reliable and consistent cortical three-dimensional organoids [125]. Additionally, another study showed that 95% of individual dorsal forebrain three-dimensional organoids had an indistinguishable collection of cell types that showed consistent developmental trajectories. The variability among three-dimensional organoids was comparable to the variability of individual endogenous human brains. Furthermore, three-dimensional organoids from different stem cell lines showed consistency in the composition of different cell types produced [126]. This successful three-dimensional organoid-to-organoid reproducibility suggested that brain three-dimensional organoids can be a tractable model. Along with advances in the recapitulation of brain development and disease modelling, significant ethical issues of possible consciousness in cerebral three-dimensional organoids have risen. With the constantly developing research in three-dimensional organoid technology, refined and advanced brain three-dimensional organoids may become conscious such as developing memories, the ability to feel pain, or self-awareness. Therefore, specific relevant research policies and overseeing bodies need to be established for this emerging field [127,128].

## 4. Conclusion

The establishment of patient-derived models faithfully reproducing normal physiology and disease pathogenesis are essential for investigating molecular mechanisms, identifying new diagnostic and prognostic biomarkers, and personalized patient treatments. Because brain three-dimensional organoids derived from individuals' iPSCs maintain the major characteristics of the developing brain with identical genetic information, the brain three-dimensional organoid system has enormous potential to pave the way for personalized medicine for NDDs.

Two-dimensional models are useful tools to evaluate NDDs, as they are easy to manage, cost-effective, and have fewer ethical considerations. Nevertheless, because of their limitations to mimic the *in vivo* environment, such as cell-to-cell and cell-to-extracellular matrix interactions, of the human brain, it has been challenging to unveil the pathology and pathogenesis of NDDs.

Brain three-dimensional organoids are self-organized three-dimensional multicellular cultures grown *in vitro*. These three-dimensional organoids can mimic the human brain to an extent, resembling the complexity of this organ *in vivo*, and can be used to study NDDs through gene and protein expression, signalling pathways. Furthermore, iPSCs-derived organoids have the potential to differentiate into diverse neuronal cell subtypes, such as motor neurons, oligodendrocytes and astroglia. Nevertheless, the lack of vasculature and microglia are now the challenge to overcome.

For this, a combination of approaches might be useful to continue adding other characteristics to the brain three-dimensional organoid, in order to fully resemble the complexity of the organ. With further advances on iPSC-derived three-dimensional organoids, larger and mature brain organoids could be achieved to continue studying the pathophysiology of NDDs.

## Data Availability

This article has no additional data.
